# The epigenetic determinants for systemic juvenile idiopathic arthritis phenotyping and treatment response

**DOI:** 10.1186/s12891-024-07702-9

**Published:** 2024-08-06

**Authors:** Doaa Mosad Mosa, Shorouk Mohsen, Mohamed Taman, Nada Khaled, Sherine Mohamed Gaafar, Mona S. Abdelhafez, Rasha Elmowafy, Marwa H. Elnagdy, Ali Sobh

**Affiliations:** 1 Department of Rheumatology & Rehabilitation, Mansoura University Hospitals, Mansoura University Faculty of Medicine, Mansoura, Egypt; 2 Department of Public Health and Preventive Medicine, Faculty of Medicine, Mansoura University, Mansoura, Egypt; 3 Department of Obstetrics and Gynecology, Mansoura University Hospital, Mansoura Faculty of Medicine, Mansoura, Egypt; 4 Department of Clinical Pathology (Hematology unit), Faculty of Medicine, Mansoura University, Mansoura, Egypt; 5 Department of Medical Microbiology and Immunology, Faculty of Medicine, Mansoura University, Mansoura, Egypt; 6 Department of Medical Biochemistry and Molecular Biology, Mansoura University Faculty of Medicine, Mansoura, Egypt; 7 Department of Pediatrics, Mansoura University Children’s Hospital, Mansoura University Faculty of Medicine, Mansoura, Egypt

**Keywords:** Systemic juvenile arthritis, microRNAs, Disease activity, Treatment response, Epigenetics

## Abstract

**Background:**

Determining the role of epigenetics in systemic juvenile idiopathic arthritis (SJIA) provides an opportunity to explore previously unrecognized disease pathways and new therapeutic targets.

**Aim:**

We aimed to identify the clinical significance of microRNAs (miRNA-26a, miRNA-223) in SJIA.

**Materials and methods:**

This cross-sectional study was conducted on a group of children with SJIA attending to pediatric rheumatology clinic, at Mansoura University Children’s Hospital (MUCH) from December 2021 to November 2022. Patient demographics, and clinical, and laboratory data were collected with the measurement of microRNAs by quantitative real-time PCR. The Mann–Whitney, Kruskal–Wallis, and Spearman correlation tests were used for variable comparison and correlations, besides the receiver operating characteristic (ROC) curve for microRNAs disease activity and treatment non-response discrimination.

**Results:**

Forty patients were included in the study. On comparison of miRNA-26a, and miRNA-223 levels to the clinical, assessment measures, and laboratory features, miRNA-26a was statistically higher in cases with systemic manifestations versus those without. Similarly, it was higher in children who did not fulfill the Wallace criteria for inactive disease and the American College of Rheumatology (ACR) 70 criteria for treatment response. Meanwhile, miRNA-223 was not statistically different between cases regarding the studied parameters. The best cut-off value for systemic juvenile arthritis disease activity score-10 (sJADAS-10) and the ability of miRNA-26a, and miRNA-223 to discriminate disease activity and treatment non-response were determined by the (ROC) curve.

**Conclusion:**

The significant association of miRNA-26a with SJIA features points out that this molecule may be preferentially assessed in SJIA disease activity and treatment non-response discrimination.

## Introduction

Systemic juvenile idiopathic arthritis (SJIA) is characterized by a spiking fever of more than 39 °C, in association with arthritis, and represents 10–15% of all JIA cases. The extra-articular manifestations described in SJIA include lymphadenopathy, hepatosplenomegaly, polyserositis, evanescent macular rash, and myocarditis [1]. Children with SJIA are also at risk for developing macrophage activation syndrome (MAS), a potentially fatal complication in 5–10% of cases [2].

SJIA is considered a polygenic disease, its pathogenesis is attributable to dysregulation of the innate immunity, with auto-inflammatory predominance compared to the other JIA subtypes. Therapies that antagonize pro-inflammatory cytokines such as interleukin-1 (IL-1) and IL-6 have promising results in the management of SJIA [3].

Variable genetic and epigenetic markers are known as risk factors in disease pathogenesis moreover, they are useful for predicting treatment outcomes, selecting the proper drug for each patient, maximizing treatment efficacy, and reducing long-term complications [4].

The rapid control of inflammation allows for avoiding structural damage and growth impairment. There are many treatment options, including disease-modifying anti-rheumatic drugs (DMARDs) and biologics [5] but there are about 35–45% of patients fail to respond. Understanding the molecular elements, such as variants in genes of therapeutic relevance, influencing treatment response in SJIA, would be important to individualize treatment strategies [6].

More than 80% of the human genome is transcribed into RNA transcripts without protein-coding potential. The epigenome encompasses many layers including DNA methylation, histone modifications, and non-coding RNAs, which interact to influence gene transcription, cell function, and disease risk [7].

MicroRNAs (miRNAs) (16–24 nucleotides) are involved in various gene regulation mechanisms through interactions with transcription factors or epigenetic modifiers [8]. Their presence in plasma and serum makes them potential non-invasive biomarkers for disease activity and progression evaluation [9]. SJIA has special clinical features and an inflammatory profile compared with other JIA subtypes [10]. Therefore, we speculated that miRNA profiling might also be different in patients with SJIA.

Previous studies signified the value of measuring miRNA in SJIA patients and concluded that they were significantly higher in active children than those in the inactive phase. They are upregulated in peripheral blood mononuclear cells (PBMCs), synoviocytes, and synovial fluid [11–13].

Determining the role of epigenetics in JIA provides an opportunity to unveil previously unrecognized disease pathways and new therapeutic molecular targets. Additionally, therapeutic approaches that correct epigenetic aberration by targeting epigenetic avenues are developing rapidly and are of growing interest in rheumatic disease. We aimed to explore the clinical significance of microRNAs (miRNA-26a, miRNA-223) in SJIA patients, evaluate the potential role of these miRNAs as diagnostic and prognostic tools, and correlate miRNAs levels with clinical features, disease activity, damage index, and medical therapies.

## Materials and methods

### Patients

This cross-sectional study was conducted on a group of children with SJIA attending to the pediatric rheumatology clinic, Mansoura University Children’s Hospital (MUCH), Mansoura, Egypt, from December 2021 to November 2022.

Sample size calculation was based on the mean (SD) of miRNA among cases with JIA retrieved from previous research [11]. Using G power version 3.1.9.4 with effect size 0.469, 2-tailed, α error = 0.05, and power = 90.0%; the total calculated sample size was 40 patients.

#### Inclusion criteria

SJIA patients were diagnosed and classified according to the International League of Associations for Rheumatology (ILAR), and in different stages of disease activity and severity [14].

#### Exclusion criteria

1. Children with other JIA subtypes or systemic autoimmune disorders.
2. Cases with other conditions that can affect the expression of these epigenetic factors as infection, diabetes mellitus, malignancies, or those with overlap syndromes.

### Data collection

Data were collected from our medical files and interpreted concerning the demographic, clinical, disease assessment parameters, and laboratory features of the disease as follow:

1. Disease assessment including: disease activity by systemic juvenile arthritis disease activity score-10 (sJADAS-10) [15], assessment of function by child- health assessment questionnaire (C-HAQ) [16], disease damage assessment by juvenile arthritis damage (JAD) index [17], inactive disease definition by Wallace criteria [18], and the American College of Rheumatology (ACR) 70 was used as a standardized measure of treatment response [19].
2. Laboratory investigations: Complete blood count (CBC), erythrocyte sedimentation rate (ESR), C-reactive protein (CRP), liver function tests, serum creatinine, and serum ferritin were obtained from the patients’ medical records as routine laboratory tests in caring for SJIA cases.

### Sample collection for miRNAs analysis

miRNAs isolation was carried out at the Medical Biochemistry and Molecular Biology Department, Mansoura Faculty of Medicine. Two milliliters of blood were collected in EDTA-containing blood collection tubes from 40 patients and used to assess the expression of miRNA-26a, and miRNA-223 by Quantitative real-time polymerase chain reaction (qRT-PCR). Blood was treated with RBCs lysis buffer, then centrifuged till separation of the white blood cells. Total RNA extraction was performed utilizing miRNeasy Mini Kit (QIAGEN, Germany) in accordance with the manufacturer′s instructions. The RNA concentration and purity were checked by Thermo Scientific NanoDrop One. Reverse transcription of 1ug of RNA was done using SensiFAST™ cDNA Synthesis Kit (Bioline, UK) on Applied Biosystems Proflex Thermal Cycler. cDNA templates were amplified using a real-time PCR instrument (Azure Cielo 6, Azure, USA).

**Fig. 1:**
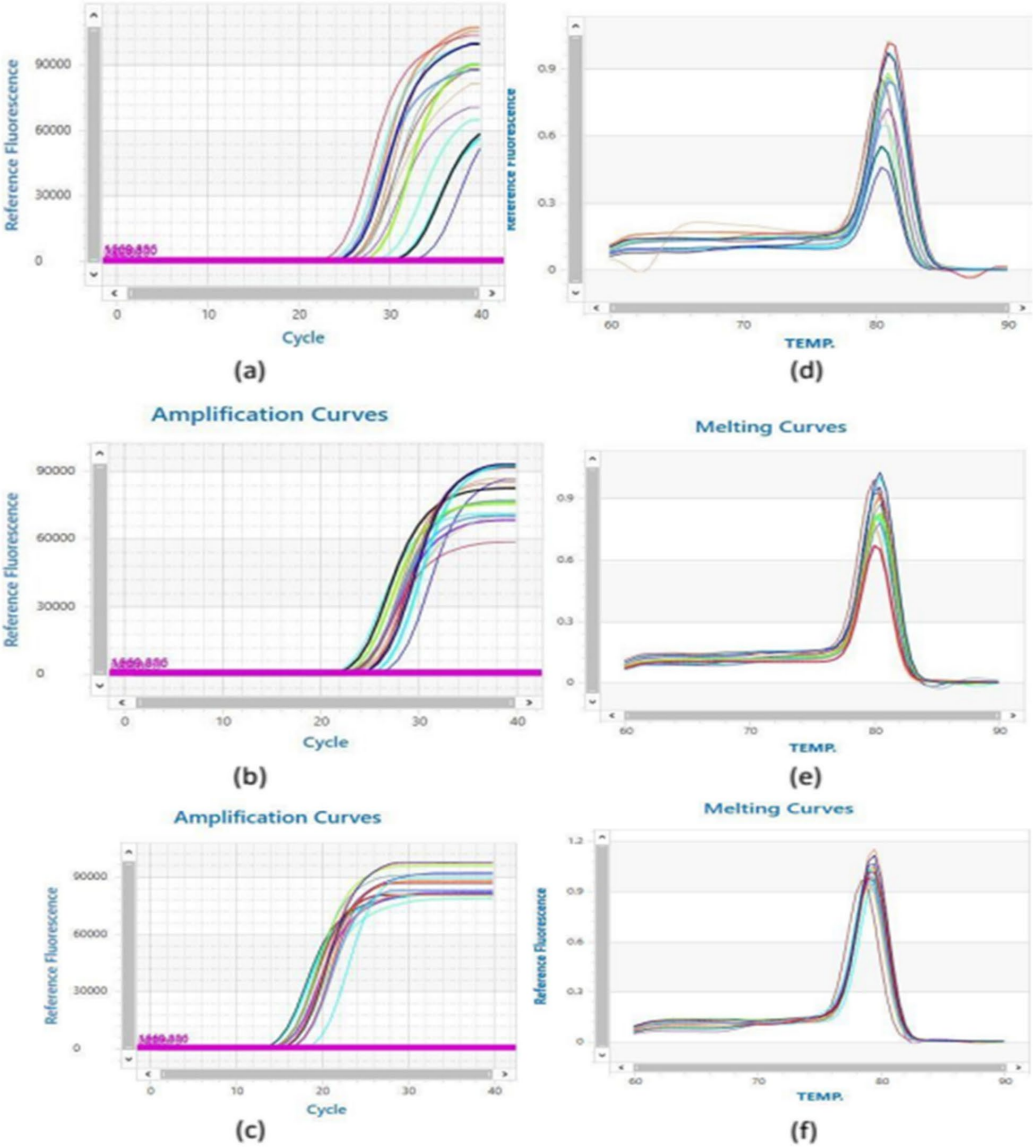
**a** Amplification plot of miRNA-26a expression. **b** Amplification plot of miRNA-223 expression. **c** Amplification plot of beta-actin expression. **d** Melting curve of miRNA-26a. **e** Melting curve of miRNA-223. **f** Melting curve of beta-actin

The amplification reaction was done in 20 μl total reaction volume [10 μl of Bioline SYBR green PCR Master Mix (Bioline, UK), 1μl of cDNA template, 2 μl (10 pmol/μl) gene primer, and 7 μl of nuclease-free water] using the following program: 95°C for 2 min, 40 cycles of 95°C for 10 s, 60 °C for 30 s. β-actin was used as an endogenous reference gene to normalize the miRNA expression levels. The sequences of the used primer pairs are supplied in Table 1. The primer sets were designated using Primer3Plus software (http://www.bioinformatics.nl/cgi-bin/primer3plus/primer3plus.cgi), and primer specificity was checked using Primer-BLAST program (NCBI/ primer-BLAST (https://www.ncbi.nlm.nih.gov/tools/primer-blast/). Primers were synthesized by Vivantis (Vivantis Technologies, Malaysia). The melting curve for each primer detected a single sharp peak that indicates the specificity of the primer (Fig. 1).

**Table 1:** The sequence of human primers used in quantitative real-time PCR analysis

Gene	Sequence	Product size	Reference Sequence
miRNA 26a	**Forward primer:** TGGCCTCGTTCAAGTAATCCA **Reverse primer:** CCCCGTGCAAGTAACCAAGA	72 bp	NR_029499
miRNA 223	**Forward primer:** CCACGCTCCGTGTATTTGAC **Reverse primer:** CCGCACTTGGGGTATTTGAC	79 bp	NR_029637.1
β-actin	**Forward:** GTGGCCGAGGACTTTGATTG **Reverse:** GTGGGGTGGCTTTTAGGATG	104 bp	NM_001101.5

*bp* base pair

Relative gene expression levels were calculated as ΔCt = Ct target gene– Ct endogenous reference gene. The fold change of gene expression was calculated according to the 2^−ΔΔCT^ method [20].

### Statistical analysis

Data were analyzed using IBM SPSS Statistics for Windows, Version 25.0. (IBM Corp, 2017). Number and percentage of the total were used for expressing categorical data, while median and interquartile range (IQR) were used to display non-normally distributed data. To compare the miRNA-26a, and miRNA-223 levels with the different clinical and laboratory features, the Mann–Whitney and Kruskal–Wallis tests were applied. The Spearman correlation was used to find correlations between non-normally distributed continuous data. The best cut-off point for sJADAS and the ability of miRNA-26a, and miRNA-223 to discriminate disease activity and treatment non-response were tested by the receiver operating characteristic (ROC) curve using MedCalc for Windows, version 14.8.1 (MedCalc Software, Ostend, Belgium). For all the above-mentioned statistical tests, the results were considered significant when *p* ≤ 0.05.

### Ethical considerations

The study was conducted in accordance with the declaration of Helsinki and informed consent was obtained from each parent before the procedures. The study was also carried out after the approval of the Institutional Research Board (IRB) of the Faculty of Medicine, Mansoura University, Egypt (R.21.03.1276).

## Results

A total of 40 patients were included in this study. Table 2 exhibits a description of demographics, clinical features, family history, and disease complications. Among our cohort, the majority were female (52.5%), whereas the median age at the disease onset and disease duration were 5.8 and 2 years, respectively. The arthritis domain (72.5%) was more predominated than systemic manifestations (62.5%). The most frequently reported complication was stunted growth (10%).

**Table 2:** Demographic data of studied SJIA patients

Variable	N (%) / Median (IQR) *N* = 40
**Age**	7.8(6–12)
**Sex**	
Male	19 (47.5)
Female	21 (52.5)
**Age of disease onset**	5.8 (4–9)
**Duration of disease (years)**	2 (0.6–3)
**BMI (Mean ± SD)**	21.1 ± 4.2
**Family history of any autoimmune rheumatic disorders**	1(2.5)
**Systemic manifestations**	25 (62.5)
**Arthritis**	29 (72.5)
**Complication**	
Stunted growth	4 (10)
Osteoporosis	1 (2.5)
Avascular necrosis	1 (2.5)
Interstitial lung diseases	1 (2.5)

*BMI* body mass index

Regarding the sJADAS-10 median, it was 21.5; meanwhile, the median of C-HAQ was 0.2. The maximum JAD-A or JAD-E was 3 which was reported in 4 and 1 of our cases, respectively. The treatment dispensed for patients was summarized in Table 3; the most frequently received combination was steroid and DMARDs (mainly methotrexate or leflunomide) (N:20, 48.8%) and the least used therapy was methotrexate with biological therapy (tocilizumab) (N:1, 2.4%). The number of patients who achieved the Wallace criteria for inactive disease and ACR 70 for treatment response was 17 and 19 children, respectively. The overall laboratory findings in our cohort are displayed in Table 3. The median of miRNA 26a was 0.16 with a range of (0.04–1.9). While the median of miRNA 223 was 0.79 with a range of (0.5–1.2).

**Table 3:** The activity parameters, functional status, laboratory findings, and medications used by SJIA patients

Variable	N (%) / Median (IQR) *N* = 40
**sJADAS-10**	21.5 (5.8–32.8)
**C-HAQ**	0.2 (0.1–0.8)
**JAD Index-A**	
0	32 (80)
2	4 (10)
3	4 (10)
**JAD-E**	
0	34 (85)
1	3 (7.5)
2	2 (5)
3	1 (2.5)
**Treatment type**	
Off-treatment	3 (7.3)
DMARDs	5 (12.2)
DMARDs + biological therapy	1 (2.4)
DMARDs + steroids	20 (48.8)
DMARDs + steroids + biological therapy	9 (22)
Steroids + biological therapy	3 (7.3)
**Treatment response by ACR 70 criteria**	19 (47.5)
**Inactive disease by Wallace criteria**	17 (42.5)
**Laboratory findings**	
Anemia N (%)	20 (50)
Leucocytosis N (%)	13 (32.5)
Thrombocytosis N (%)	13 (32.5)
Elevated liver enzyme N (%)	6 (15)
ESR Median (IQR) (mm/hr)	35 (20–91.3)
CRP Median (IQR) (mg/dL)	14 (5–57)
Serum ferritin Median (IQR) (ng/mL)	95 (30.5–139.5)
**miRNA 26a**	0.16 (0.04–1.9)
**miRNA 223**	0.79 (0.5–1.2)

DMARDs include: methotrexate and leflunomide, and biological therapy includes: tocilizumab, etanercept, and sarilumab

*sJADAS-10* systemic juvenile arthritis disease assessment score-10, *C-HAQ* child-health assessment questionnaire, *JAD index-A* juvenile arthritis damage-articular, *JAD-E* juvenile arthritis damage-extra-articular, *DMARDs* disease modifying anti-rheumatic drugs, *ACR* American College of Rheumatology, *ESR* erythrocyte sedimentation rate, *CRP* C-reactive protein

On comparison of miRNA-26a, and miRNA-223 levels to the clinical, assessment measures, and laboratory features, only miRNA-26a was statistically higher in cases with systemic manifestations versus those lack thereof (*p* < 0.05) and patients with higher sJADAS-10 (> 21 points) (*p* < 0.05). Similarly, it was higher in children who did not fulfill the Wallace criteria for inactive disease (*p* < 0.05) or ACR 70 criteria for treatment response (*p* < 0.05). Meanwhile, miRNA-223 was not statistically different between cases regarding the studied parameters (Table 4).

**Table 4:** Comparison of miRNA-26a, and miRNA-223 levels according to the clinical and laboratory features

Variable	miRNA-26a	*P* value	miRNA-223	*P* value
	Median (IQR)		Median (IQR)	
**Systemic manifestations**				
No	0.007 (0.004–0.14)	0.04^*^	1.1 (0.56–1.4)	0.5
Yes	0.12 (0.06–0.4)		0.85 (0.48–1.3)	
**Arthritis**				
No	0.34 (0.003–0.13)	0.1	1.2 (0.6–2.2)	0.4
Yes	0.07 (0.008–0.3)		0.9 (0.48–1.3)	
**Disease duration**				
** < 1 year**	0.13(0.06–0.3)	0.2	0.98(0.18–1.22)	0.5
** ≥ 1 year**	0.065(0.004–0.18)		0.9(0.5–1.4)	
**Complications**				
No	0.07 (0.004–0.2)	0.9	0.85 (0.48–0.32)	0.3
Yes	0.13 (0.005–0.16)		1.19 (0.65–2.9)	
**sJADAS-10** b				
** ≤ 21 (inactive disease)**	0.03 (0.004–0.15)	0.04^*^	0.98 (0.5–1.4)	0.7
** > 21 (active disease)**	0.13 (0.06–0.4)		0.9 (0.5–1.2)	
**JAD Index-A**				
0	0.07 (0.005–0.18)	0.9	0.85 (0.47–1.3)	0.5
≥ 1	0.14 (0.003–0.3)		1.15 (0.7–2.6)	
**JAD-E**				
0	0.07 (0.004–0.19)	0.4	0.85 (0.49–1.36)	0.4
≥ 1	0.14 (0.005–2.25)		1.19 (0.6–1.7)	
**Inactive disease by Wallace criteria**				
No	0.12 (0.06–0.3)	0.045^*^	0.86 (0.5–1.34)	0.9
Yes	0.007 (0.004–0.15)		0.85 (0.5–1.73)	
**Anemia**				
No	0.07 (0.004–0.14)	0.2	0.98 (0.33–1.36)	0.9
Yes	0.1 (0.007–0.44)		0.82 (0.5–1.3)	
**Leukocytosis**				
No	0.07 (0.004–0.27)	0.9	1.1 (0.56–1.4)	0.3
Yes	0.07 (0.047–0.18)		0.77 (0.09–1.29)	
**Thrombocytosis**				
No	0.07 (0.004–0.18)	0.1	1.1 (0.53–1.95)	0.2
Yes	0.06 (0.02–0.26)		0.77 (0.28–1.28)	
**Elevated liver enzyme**				
No	0.07 (0.004–0.22)	0.9	0.86 (0.55–1.33)	0.7
Yes	0.1 (0.05–0.24)		0.87 (0.07–1.82)	
**Treatment response by ACR 70 criteria**				
No	0.13 (0.06–0.4)	0.07^*^	1.19 (0.6–1.68)	0.2
Yes	0.008 (0.004–0.16)		0.7 (0.5–1.2)	
**Treatment type**				
Off-treatment	0.07(0.003–0.07)	0.2	1.95(1.4–1.95)	0.1
DMARDs	0.004(0.003–0.06)		0.68(0.56–0.85)	
DMARDs + biological therapy	0.004(0.004–0.004)		0.27(0.27–0.27)	
DMARDs + steroids	0.14(0.06–0.3)		0.98(0.47–1.19)	
DMARDs + steroids + biological therapy	0.07(0.004–0.4)		1.27(0.56–2.7)	
Steroids + biological therapy	0.07(0.003–0.07)		0.09(0.08–0.09)	

*b* SJDAS-10 cut-off based on ROC curve analysis. *sJADAS-10* systemic juvenile arthritis disease assessment score-10, *JAD index-A* juvenile arthritis damage-articular, *JAD-E* juvenile arthritis damage-extra-articular, *ACR* American College of Rheumatology, DMARDs include: methotrexate and leflunomide, and biological therapy include: tocilizumab, etanercept, and sarilumab. The Mann–Whitney and Kruskal–Wallis tests were used for comparison

^*^p < 0.05 is statistically significant

miRNA 26a was found to be positively correlated to sJADAS-10 (*p* < 0.05), C-HAQ (*p* < 0.05), both ESR and CRP (*p* < 0.05) but was not correlated to JAD-A, JAD-E, or serum ferritin. On the other hand, miRNA 223 was not correlated to any of the disease assessment measures or laboratory findings (Table 5).

**Table 5:** Correlation between miRNA-26a, miRNA-223 levels and characteristics of SJIA patients

	**miRNA-26a**		**miRNA-223**	
	**r**	***P* value**	**r**	***P* value**
**sJADAS-10**	0.3	0.006^*^	-0.07	0.7
**C-HAQ**	0.3	0.04^*^	0.06	0.7
**JAD Index-A**	0.02	0.9	0.1	0.4
**JAD-E**	0.1	0.4	0.1	0.5
**ESR**	0.3	0.028^*^	0.02	0.9
**CRP**	0.3	0.03^*^	0.03	0.9
**Serum ferritin**	0.2	0.4	-0.1	0.6

The Spearman correlation test was used

*sJADAS-10* systemic juvenile arthritis disease assessment score-10, *C-HAQ* child-health assessment questionnaire, *JAD index-A* juvenile arthritis damage-articular, *JAD-E* juvenile arthritis damage-extra-articular, *ESR* erythrocyte sedimentation rate, *CRP* C-reactive protein

^*^p < 0.05 is statistically significant

**Fig. 2:**
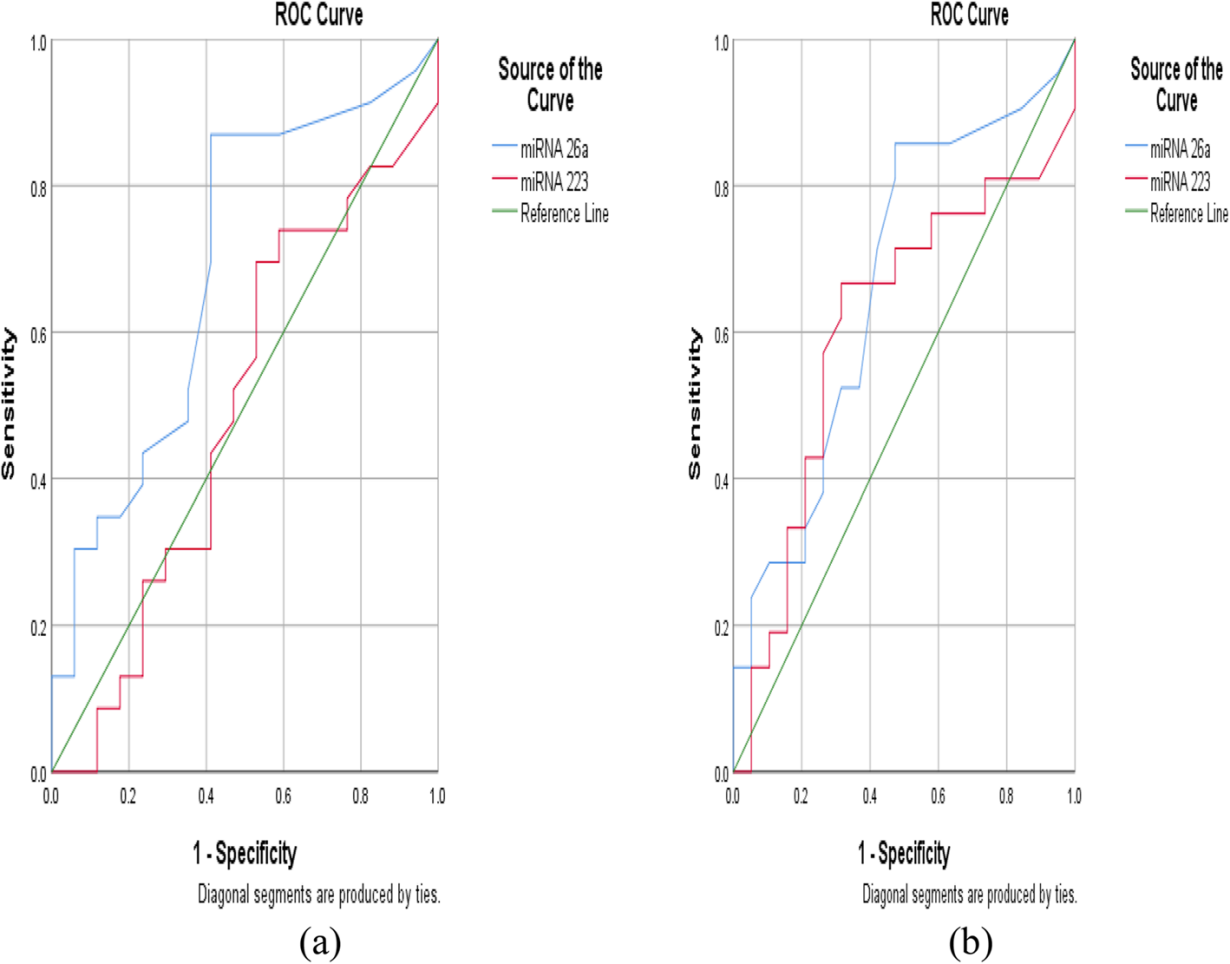
**a** ROC curve analysis of miRNA-26a and miRNA-223 to discriminate disease activity **b** ROC curve analysis of miRNA-26a and miRNA-223 to discriminate treatment non-response

The ROC curve detected that the best cut-off of miRNA 26a to discriminate disease activity is greater than or equal to 0.008 with a sensitivity of 86.9% and specificity of 58.8%. While its cut-off to differentiate treatment non-responders is greater than or equal to 0.008 with a sensitivity of 85.7% and specificity of 52.6%. The miRNA 223 cut-off of 0.68 or more attains a sensitivity of 52.9% and specificity of 30.4% to distinguish disease activity. However, it had a sensitivity of 66.6% and specificity of 68.4% to discriminate treatment non-responders at a cut-off of 0.85 (Table 6) (Fig. 2).

**Table 6:** ROC curve analysis for miRNA-26a, and miRNA-223 levels to discriminate disease activity and treatment non-response

	**Cut off value**	**Sensitivity**	**Specificity**	**AUC**
**Disease activity**^a^				
**miRNA-26a**	≥ 0.008	86.96	58.82	0.688
**miRNA-223**	≥ 0.68	52.94	30.43	0.51
**No treatment response by ACR 70 criteria**				
**miRNA-26a**	≥ 0.008	85.7	52.63	0.67
**miRNA-223**	≥ 0.85	66.67	68.42	0.6

*ACR* American College of Rheumatology

^a^Disease activity based on sJADAS-10 > 21

## Discussion

The miRNAs have various immunomodulatory functions and their expression increases in the synovial fluid in cases with chronic arthritis. Circulatory miRNAs may represent valid biomarkers for SJIA differentiation due to their stability and sensitivity. Hence, specialized studies on the immunologic print of miRNAs in the SJIA patients’ characterization are particularly important [21].

The findings of this study supported the results of previously published reports that confirmed the potential value of miRNA 26a and miRNA 223 measurement in SJIA [11, 13, 22, 23].

miRNA-26a is involved in the regulation of cellular proliferation and differentiation [24]. Moreover, it has an anti-inflammatory effect as it inhibits osteoclast differentiation, blocks the action of Toll-like receptors on macrophages surface, antagonizes the effect of inflammatory cytokines, and has anti-angiogenesis effects [25–27].

In this analysis, miRNA-26a was statistically higher in cases with systemic manifestations when compared to those without these presentations and in patients with higher sJADAS-10. Likewise, it was higher in cases that did not fulfill the Wallace criteria for inactive disease or ACR 70 criteria of treatment response. Sun et al. [11] focused on miRNA-26a and miR-145 expression in SJIA and they found that the miRNA-26a was preferentially upregulated in SJIA patients compared to oligo-articular, poly-articular, enthesitis-related arthritis JIA subtypes as well as cases with systemic lupus erythematosus (SLE), IgA vasculitis, and Kawasaki disease (KD).

Further, when they did the ROC analysis, the AUC showed the ability of miR-26a to discriminate SJIA patients from healthy controls [11]. Another study confirmed that this microRNA was elevated in active SJIA children and can be used as a marker of disease activity [28]. Moreover, high expression of miRNA-26a was also detected in rheumatoid arthritis (RA) patients compared to healthy controls [29].

miRNA 26a was found to be correlated to sJADAS-10 and C-HAQ but was not correlated to damage index either articular or extra-articular in our cohort. On the other hand, when correlated previously to other variables like acute phase reactant, and swollen, or tender joint count, there was no correlation observed [11] despite being positively correlated to IL-6 as a marker of active disease in SJIA [30].

miRNA 223 is expressed in CD4 T-cells and is an important modulator of myeloid cell differentiation including osteoclasts that are involved in bone erosion [31]. The upregulation of miRNA 223 in T lymphocytes plays a central role in the development of local joint inflammatory process rather than systemic effects [32].

We found that miRNA-223 was not statistically different between cases concerning, the clinical, assessment parameters of disease activity, damage indices, or laboratory findings nor did correlate to the acute phase reactants or disease assessment measures. Ma et al. [22] could not detect any strong correlation between studied biological or clinical features of JIA patients and miRNA 223 expression, however, it was up-regulated in poly-articular JIA patients compared to healthy subjects. This miRNA could be more correlated to the matrix metalloproteinase-3 in polyarthritis and oligo-articular subtypes rather than SJIA as concluded previously [12, 33].

Studies in RA cases did not show any difference between miRNA 223 in patients and healthy control and its levels seem to be moderately elevated in the peripheral mononuclear cells than local sites of inflammation. In addition, it was inversely correlated to tender joint counts [34].

Nevertheless, it has been reported that miRNA 223 is upregulated in active SJIA compared to inactive cases or healthy controls and it was also correlated to ESR in SJIA and poly-articular JIA [13]. Further, miRNA 223 was elevated in the RA cohort compared to healthy subjects and implicated in bone erosions [35]. It was also reported to be correlated to CRP levels in RA cases [36]. Castro-Villegas et al. [37] concluded that miR-223 may serve as a predictor of patient’s response to the biological/DMARDs therapies in RA cases.

Indeed, there are conflicting results about the value of miRNA 223 in inflammatory arthritis. However, the pathogenesis of SJIA is different than other subtypes; it is a unique subtype characterized by innate immunity dysfunction. miRNA 223 is a reflection of lymphocyte activity in autoimmune arthritis and it seems that the magnitude of the local production of this microRNA is higher than its systemic expression [34]. Furthermore, this category comprises mechanisms that are more diverse than RA as JIA cases still undergo skeletal and immune system maturation.

In ROC curve analysis, we provide for the first time the best cut-offs of both miRNA 26a and miRNA 223 for disease activity and treatment non-responsiveness discretion in SJIA. Nevertheless, more rigorous studies with prospective cohort studies on larger series are essential to validate our findings. Our study’s limitations are that it was a cross-sectional research, small sample size that limits the regression analysis testing, no quantification of miRNAs in synovial fluid that is, certainly, more invasive than just obtaining blood samples, and financial constraints limit more miRNAs molecules analysis.

## Conclusion

This work suggests that circulatory miRNAs specifically miRNA 26a could serve as potential biomarkers for SJIA assessment of disease activity and distinguish treatment non-responders in the epigenetic milieu.

## Data Availability

All data generated during this study are in this published article.
